# Repatriation of an old fish host as an opportunity for myxozoan parasite diversity: The example of the allis shad, *Alosa alosa* (Clupeidae), in the Rhine

**DOI:** 10.1186/s13071-016-1760-6

**Published:** 2016-09-15

**Authors:** Hannah Wünnemann, Astrid Sybille Holzer, Hana Pecková, Pavla Bartošová-Sojková, Ulrich Eskens, Michael Lierz

**Affiliations:** 1 Clinic for Birds, Reptiles, Amphibians and Fish, Justus Liebig University, Frankfurter Str. 91, Giessen, 35392 Germany; 2 Institute of Parasitology, Biology Centre of the Czech Academy of Sciences, Branišovská 31, České Budějovice, 37005 Czech Republic; 3 Faculty of Science, University of South Bohemia, Branišovská 31, České Budějovice, 37005 Czech Republic; 4 The Hessen State Laboratory, Schubertstraße 60, Giessen, 35392 Germany

**Keywords:** Host reintroduction, *Alosa alosa*, Parasite population structure, *Hoferellus alosae* n. sp., Myxozoa, Diversity, SNPs

## Abstract

**Background:**

Wildlife repatriation represents an opportunity for parasites. Reintroduced hosts are expected to accumulate generalist parasites via spillover from reservoir hosts, whereas colonization with specialist parasites is unlikely. We address the question of how myxozoan parasites, which are characterized by a complex life-cycle alternating between annelids and fish, can invade a reintroduced fish species and determine the impact of a *de novo* invasion on parasite diversity. We investigated the case of the anadromous allis shad, *Alosa alosa* (L.), which was reintroduced into the Rhine approximately 70 years after its extinction in this river system.

**Methods:**

We studied parasites belonging to the Myxozoa (Cnidaria) in 196 allis shad from (i) established populations in the French rivers Garonne and Dordogne and (ii) repatriated populations in the Rhine, by screening the first adults returning to spawn in 2014. Following microscopical detection of myxozoan infections general myxozoan primers were used for SSU rDNA amplification and sequencing. Phylogenetic analyses were performed and cloned sequences were analyzed from individuals of different water sources to better understand the diversity and population structure of myxozoan isolates in long-term coexisting *vs* recently established host-parasite systems.

**Results:**

We describe *Hoferellus alosae* n. sp. from the renal tubules of allis shad by use of morphological and molecular methods. A species-specific PCR assay determined that the prevalence of *H. alosae* n. sp. is 100 % in sexually mature fish in the Garonne/Dordogne river systems and 22 % in the first mature shad returning to spawn in the Rhine. The diversity of SSU rDNA clones of the parasite was up to four times higher in the Rhine and lacked a site-specific signature of SNPs such as in the French rivers. A second myxozoan, *Ortholinea* sp., was detected exclusively in allis shad from the Rhine.

**Conclusions:**

Our data demonstrate that the *de novo* establishment of myxozoan infections in rivers is slow but of great genetic diversity, which can only be explained by the introduction of spores from genetically diverse sources, predominantly via straying fish or by migratory piscivorous birds. Long-term studies will show if and how the high diversity of a *de novo* introduction of host-specific myxozoans succeeds into the establishment of a local successful strain in vertebrate and invertebrate hosts.

**Graphical Abstract:**

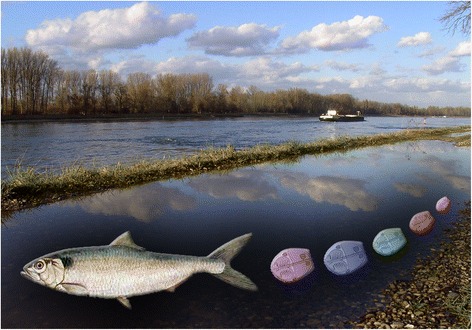

**Electronic supplementary material:**

The online version of this article (doi:10.1186/s13071-016-1760-6) contains supplementary material, which is available to authorized users.

## Background

The allis shad, *Alosa alosa* (L.) is an anadromous clupeid fish whose original distribution covered the area from the coast of southern Scandinavia to that of northwestern Africa. This species has a pelagic marine existence but upon maturation (4 to 6 years) migrates to spawn in the higher middle watercourse of rivers [1]. The populations of allis shad decreased severely by the middle of the 20th Century and are regarded as endangered on a European level. A combination of anthropogenic factors, such as the construction of dams on rivers that prevent spawning migrations, the destruction of spawning grounds, over-fishing and increasing pollution were considered to be causal [2, 3]. Residential populations of allis shad were likewise extinct in the Rhine ecosystem by the middle of the 20th Century [4, 5] until their repatriation in the course of the EU-LIFE project “The reintroduction of Allis shad (*Alosa alosa*) in the Rhine system” (2007–2010) and the follow-on EU-LIFE+ project “Conservation and Restoration of the Allis shad in the Gironde and Rhine watersheds” (2011–2015). Within these projects, allis shad brood stock from a natural population in France were spawned in captivity and 10.66 million larvae reared in aquaculture facilities were released into the Rhine system, between 2008 and 2014 [6, 7]. Adult shad first returned to spawn in the Rhine six years after the release of larvae in these waters [7].

Wildlife repatriation after long periods of absence offers a great opportunity for obtaining real time data on the recolonization of hosts with parasites and for understanding local species diversity. Whilst generalist parasites can be obtained from reservoir hosts in a relatively short period of time, colonization with specialist parasites is dependent on the contact with members of established fish populations elsewhere [8]. Allis shad shows natal site fidelity which provides fitness benefits due to local adaption [9, 10]. However, some degree of straying has been observed, especially between neighboring populations [11].

Myxozoans are morphologically extremely reduced cnidarian parasites with complex life-cycles that require an invertebrate (predominantly annelid) definitive host and a vertebrate (mainly fish) intermediate host [12, 13]. In the present study, allis shad originating from natural populations in the Dordogne/Garonne river systems were found infected with a myxozoan species belonging to the genus *Hoferellus* Berg, 1898 in the renal tubules and collecting ducts of the kidney. In cyprinids, *Hoferellus* spp. show extreme host specificity [14]. Specialist parasites with an indirect life-cycle can only establish if (i) all hosts required for completion of the life-cycle are present and (ii) one of the hosts becomes infected in a given habitat. In this study, we describe *Hoferellus alosae* n. sp. from *Alosa alosa* and investigate parasite SSU rDNA clone diversity in established host-parasite populations in the Dordogne/Garonne watershed and in the first infected hosts returning to the Rhine system for spawning. We address the questions of genetic diversity of newly establishing myxozoan specialist parasite populations and the potential sources of infection.

## Methods

### Origin of fish and diagnostic methods

**Fig. 1:**
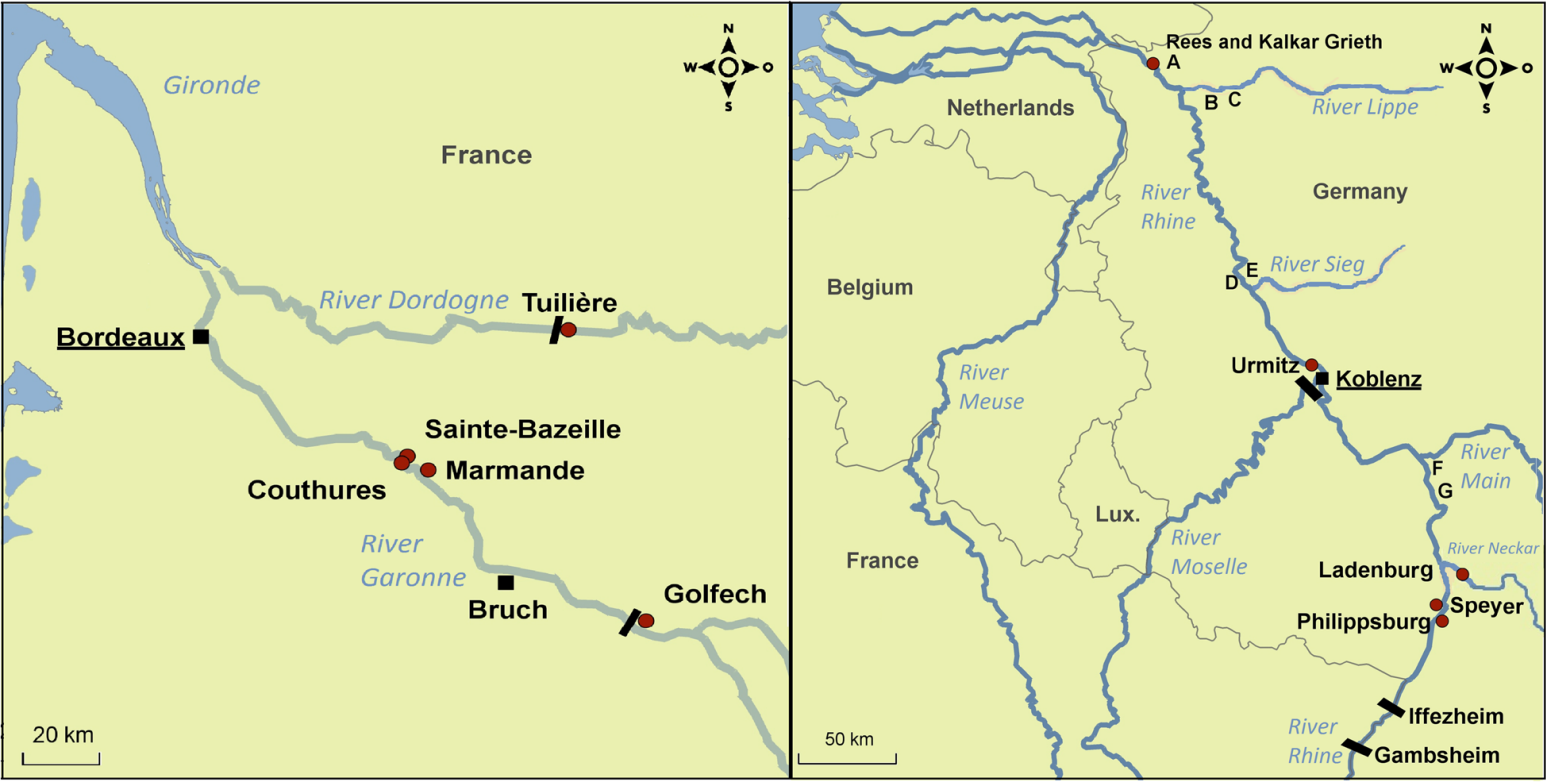
Map of France and Germany showing the watersheds studied (Dordogne, Garonne and Rhine), stocking locations (A-G, see Hundt et al. [6], sampling places (*red dots*), dams (*black bars*)

During 2012–2014, allis shad were obtained from well-established natural populations of two large rivers in south-western France, the Dordogne and the Garonne watersheds, which merge into a macrotidal estuary, the Gironde that empties into the Atlantic (Fig. 1). In the Rhine (Germany), reintroduced allis shad first returned in 2014, when adults and newly-established young-of-the-year populations were detected and sampled. In the present study, 196 wild allis shad were analyzed for myxozoan infections. The wild fish populations were represented by 110 adults and 23 young-of-the-year caught in the Garonne/Dordogne system in France, as well as 9 adults and 54 young-of-the-year from the Rhine in Germany (Fig. 1, Additional file 1: Table S1). Young-of-the-year were directly frozen or fixed in 100 % ethanol after capture. Adult allis shad caught in the Rhine were frozen immediately or necropsied within 24 h after capture. The captive brood stock, which originated from the Garonne and the Dordogne, was reared in two 10 m^3^ tanks for about 1 month at a breeding facility in Bruch, France, as part of the reintroduction program. Allis shad that died during this time period or fish euthanized at the end of the reproduction period were analyzed during the present study (Additional file 1: Table S1). Freshly dead or moribund fish were frozen or analyzed immediately on site. The diagnostic methods were dependent on the quality and quantity of available material. All freshly dead and sacrificed fish were sectioned using sterile instruments and a complete bacteriological, mycological, virological and parasitological screening including histology was performed within the framework of the project. For the study of *Hoferellus* spp. infections, squash preparations of the kidney were examined and screened using light microscopy (Zeiss, Axiostar plus, Oberkochen, Germany). Furthermore, 125 samples of anterior kidney were fixed in 5 % formalin as well as 54 whole allis shad in Bouin’s solution (Sigma-Aldrich, Taufkirchen, Germany). The Bouin-fixed samples were decolorized over 24 h [15] and afterwards dehydrated in an alcohol series together with the formalin-fixed kidney samples, transferred to xylene and embedded in paraffin at the Hessen State Laboratory, Germany. Paraffin blocks were sectioned (4 μm), stained with haematoxylin and eosin and analyzed using a Leica microscope (Leica DM 2500, Leica Mikrosysteme Vertrieb GmbH, Wetzlar, Germany).

### Parasite morphology

Kidneys from 14 freshly dead or euthanized adult allis shad of the donor population (France) were transferred into a 1.5 ml collection tube with 500 μl distilled water containing 100 U/ml Penicillin-Streptomycin solution. The material was sent to the Biology Centre of the Czech Academy of Sciences and analyzed immediately. Plasmodia and spores were examined on an Olympus BX51 microscope equipped with an Olympus DP72 digital camera. Measurements of spores (*n* = 25) follow the guidelines by Lom & Arthur (1989) [16] and were taken on digital images, using ImageJ v.1_44p (Wayne Rasband, http://imagej.nih.gov/ij).

### SSU rDNA sequence analyses and genetic variability between sites

Kidney samples of 100 allis shad (Additional file 1: Table S1) frozen at -20 °C or fixed in 100 % ethanol were used for molecular analyses. Kidneys were removed from ethanol and briefly dried on a paper towel. Thereafter they were placed in TNES urea buffer [17] and DNA was extracted using proteinase K and a simplified phenol-chloroform extraction method [18]. Two partial, overlapping myxozoan SSU rDNA sequences were amplified using nested PCR assays. Universal eukaryotic primers ERIB1 and ERIB10 [19] were used in the first round. The resulting PCR product was used in two nested PCRs, employing myxozoan-specific primers: (i) MyxGP2F [20] and ACT1R [21]; and (ii) Myxgen4F [22] and ERIB10 (see above). The following PCR cycling conditions were used: 95 °C for 3 min, thereafter 30 cycles of 94 °C for 1 min, 60 °C [first round PCR)/58 °C (nested PCRs) for 1 min, 68 °C for 2 min (first round PCR)/1 min (nested PCRs)], followed by a final elongation step at 68 °C for 10 min. PCRs were performed in 10 μl reactions using Titanium Taq DNA polymerase. PCR products were visualized on 1 % agarose gels stained with ethidium bromide, purified using a Gel/PCR DNA Fragments Extraction Kit (Geneaid Biotech Ltd., New Taipei City, Taiwan) and sequenced commercially (https://www.seqme.eu/en/). Almost complete SSU rDNA sequences of *H. alosae* were obtained from four fish each from the Garonne and the Dordogne whereas the sequences obtained from infected fish from the Rhine showed double peaks in the variable sections of the SSU rDNA, indicating mixed infections. Nested PCR products of these samples were subsequently cloned into the pDrive Vector (PCR Cloning Kit, Qiagen, Hilden, Germany) and plasmid DNA was isolated using the High Pure Plasmid Isolation Kit (Roche Applied Science, Penzberg, Germany). Twelve clones per nested PCR product (2 overlapping partial SSU rDNA amplicons, see above) per infected fish (2 individuals) from the Rhine were sequenced and analyzed (fish individuals indicated in Additional file 1: Table S1).

In order to estimate SSU rDNA genetic diversity and compare it between *Hoferellus* isolates from different fish and rivers, nested PCR products (primers MyxGP2F and ACT1R) were cloned as described above. In total, six clones per fish were sequenced from three allis shad from the Dordogne, three shad from the Garonne and two infected specimens from the Rhine. Clones were obtained from adult shad as infection was only detected in adults in the Rhine. However, to determine differences in parasite diversity between the riverine young-of-the-year and adults returning from the sea to the same river, the same number of clones (3 fish, 6 clones each) was obtained from juveniles in the Garonne. Sequences were aligned in Geneious v8.1.3. (http://www.geneious.com, [23]) using the MAFFT v7.017 algorithm [24] and the L-INS-i method, with a default gap opening penalty (–op = 1.53) and gap extension penalty (–ep = 0.0). The number and position of single nucleotide changes and of polymorphic sites was noted and the divergence calculated.

### Phylogenetic analyses

The SSU rDNA sequences obtained for the *Hoferellus* isolates from the three rivers as well as that of an *Ortholinea* sp. SSU rDNA sequence from allis shad kidneys from the Rhine were aligned with 21 ingroup taxa, which were selected on the basis of their close relatedness (BLAST result on GenBank) and to represent all sub-clades within the “freshwater myxosporean clade” *sensu* Fiala [25]. Basal freshwater myxosporeans *Myxidium lieberkuehni* Biitschli, 1882 and *Chloromyxum legeri* Tourraine, 1931 were used as outgroup taxa. Sequences were aligned as stated above and maximum parsimony (MP) analysis was performed in PAUP* v4.b10 [26], using a heuristic search with random taxa addition, the ACCTRAN option, TBR swapping algorithm, all characters treated as unordered and gaps treated as missing data. Maximum likelihood (ML) analysis was performed in RAxML v7.2.8 [27] using the GTR + Γ model. Clade support values were calculated from 1000 bootstrap replicates with random sequence additions for both MP and ML analyses. Bayesian inference analysis (BI) was performed in MrBayes v3.2.2 [28] implemented in Geneious and using the GTR + Γ + I model. MrBayes was run to estimate posterior probabilities over 1,100,000 generations via 2 independent runs of 4 simultaneous Markov Chain Monte Carlo (MCMC) algorithms with every 200th tree saved. ‘Burn-in’ was set to 100,000 generations.

### Diagnostic PCR assay

In order to estimate true prevalences of *Hoferellus* infections in allis shad we designed a diagnostic PCR assay on the basis of specific nucleotide differences in highly variable regions of the SSU rDNA gene region. Primers HaloF (5'-CTT TGC GGT TTA CCC CAG AGG-3') and HaloR (5'-AAT TTC GAC GCC CAT AGT TGC-3') were used in PCR (see cycling protocol above) using 56 °C as annealing temperature and 40 s for elongation. The resulting 865 bp PCR product was sequenced from 15 kidney isolates including all rivers (see Additional file 1: Table S1). Specificity of the PCR assay was tested on DNA isolates of phylogenetically related myxosporeans *H. cyprini* (Doflein, 1898), *H. carassii* Akhmerov, 1960, *Hoferellus* sp., *H. anurae* Mutschmann, 2004, *H. gnathonemi* Alama-Bermejo, Jirků, Kodádková, Pecková, Fiala & Holzer, 2016, *Ortholinea orientalis* (Shul’man & Shul’man-Albova, 1953) and *Ortholinea* sp. (present study). All samples obtained in this study were screened for potential *H. alosae* n. sp. infection.

### Scanning electron microscopy

The spores used for scanning electron microscopy were gently spun and pipetted onto filter paper (Millipore Millex-HV size 0.45 μl). The filter paper was stuck on a stub using Tissue-Tek and rapidly frozen (< 10^-3^ K/s) in slushy nitrogen. After freezing, the sample was transferred to a high vacuum preparation chamber (ALTO 2500, Gatan). The surface of the sample was sublimated at -95 °C, for 1 min. After sublimation, the sample was coated with a mixture of platina and paladium at a temperature of -135 °C. The coated sample was analyzed on a Field Emission Scanning Electron Microscope (JSM-7401 F, JEOL). Images were obtained at an accelerating voltage of 1 kV using GB high mode.

## Results

**Phylum Cnidaria Hatschek, 1888**

**Class Myxosporea Bütschli, 1881**

**Order Bivalvulida Schulman, 1959**

**Suborder Variisporina Lom & Noble, 1984**

**Family Sphaerosporidae Davis, 1917**

**Genus*Hoferellus*Berg, 1898**

***Hoferellus alosae*n. sp.**

***Type-host*:***Alosa alosa* (L.) (Actinopterygii: Clupeiformes: Clupeidae), allis shad.

***Type-locality*:** River Garonne, France (mainly 44°06'33"N, 0°51'14"E).

***Other localities*:** River Dordogne, France (mainly 44°50'42"N, 0°37'59"E) and River Rhine, Germany (49°04'09"N, 8°25'55"E and 49°24'19"N, 8°29'40"E); for additional sites see Additional file 1: Table S1.

***Type-material*:** Unstained spores, fixed for 1 h in neutral buffered formalin, washed and mounted in glycerol-gelatine; ethanol-fixed infected kidney tissue of *A. alosa*, DNA extracted from infected kidney tissue stored at -80 °C; 2 histological slides stained with haematoxylin and eosin are deposited in the Collection of the Laboratory of Fish Protistology, Institute of Parasitology, Biology Centre of the Czech Academy of Sciences, České Budějovice, Czech Republic (record number: IPCAS Prot 34; collection curator: Miloslav Jirků, miloslav.jirku@seznam.cz).

***Location of sporogonic stages*:** Kidney tubules (predominantly in anterior kidney), ureters and urinary bladder, exceptionally in Bowman’s capsules.

***Prevalence*:** Determined by PCR. Garonne (2012–2014): adults 100 % (33/33), young-of-the-year 63.6 % (14/22); Dordogne (2013): adults 100 % (10/10); Rhine (2014): adults 22.2 % (2/9), young-of-the-year 0 % (0/26).

***Representative DNA sequences*:** Three SSU rDNA sequences are submitted to the GenBank database under accession numbers KU301050–KU301052.

***ZooBank registration*:** To comply with the regulations set out in article 8.5 of the amended 2012 version of the *International Code of Zoological Nomenclature* (ICZN) [29], details of the new species have been submitted to ZooBank (www.zoobank.org). The Life Science Identifier (LSID) of the article is urn:lsid:zoobank.org:pub: 15E34957–2C16-43E8-B374-845148395D1B. The LSID for the new name *Hoferellus alosae* is urn:lsid:zoobank.org:act: 230BD64C-E2C6-41D6-8DFC-19DC4362F237.

***Etymology*:** Species name ‘*alosae*’ refers to the species name of the host *Alosa alosa*.

### Description

**Fig. 2:**
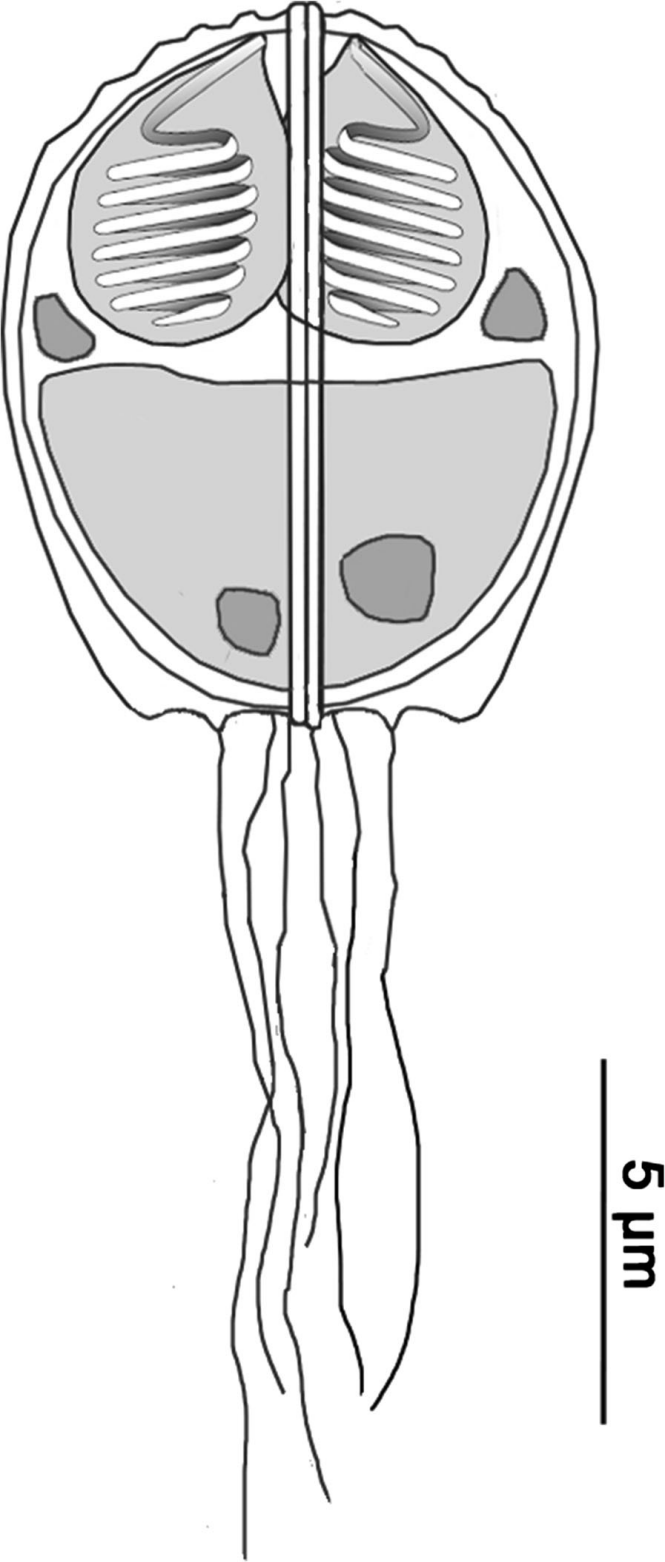
Schematic line drawing of *Hoferellus alosae* n. sp. ex *Alosa alosa*

**Fig. 3:**
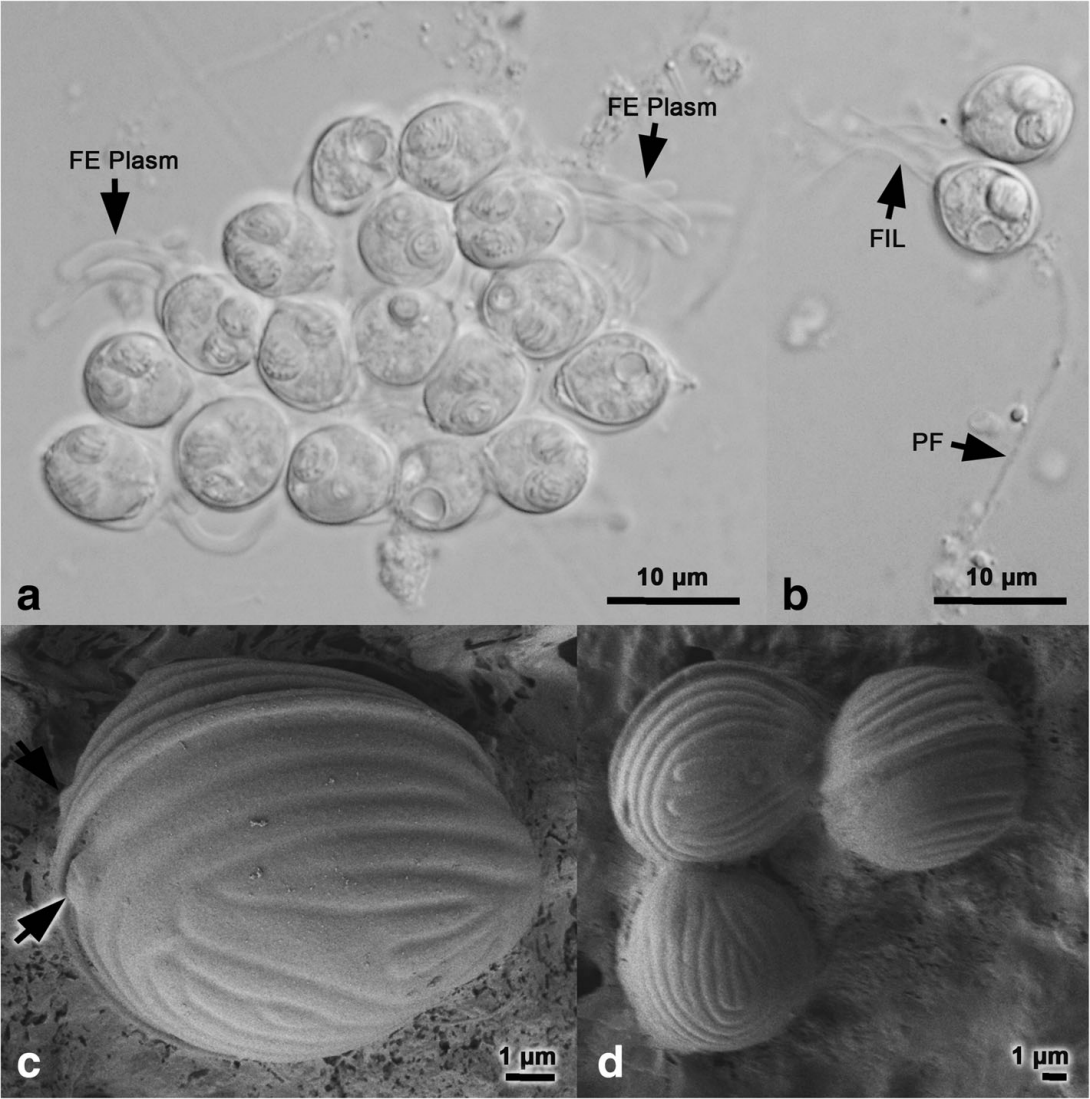
Morphological characteristics of *Hoferellus alosae* n. sp. spores and plasmodia. Light microscopy photomicrographs of **a** polysporous plasmodium with finger-like surface extensions (*FE Plasm*) and **b** spores with posterior filaments (*FIL*), one polar capsule with extruded polar filament (*PF*). **c**, **d** Scanning electron micrographs of spore surface showing suture between valves and longitudinal ridges and their characteristic pattern in the center of each valve. The *arrow* in **c** indicates capsular openings at the apical pole of the spore

***Mature spore***. Mature spores subspherical in valvular view, ellipsoidal in sutural view, posteriorly rounded, measuring 9.1–10.3 (9.7 ± 0.4) μm in length, 7.7–9.2 (8.4 ± 0.5) μm in width, and 7.2–8.3 (7.7 ± 0.3) μm in thickness (*n* = 25 spores). Valves thickened at posterior end of spore, with 2 distinct but relatively small posterior valve projections (Fig. 2), occasionally with 3 to a maximum of 7 posterior filaments measuring 5–22 μm (Figs. 2 and 3b). Sutural line between valve cells straight, well marked; valves with 12 longitudinal ridges parallel to suture line, bifurcating in center of each valve, forming a distinct pattern (Fig. 3c, d). Polar capsules 2, equally sized, subspherical, 3.5–4.4 (4.0 ± 0.2) μm long, 2.4–3.6 (3.0 ± 0.3) μm wide (*n* = 25 spores). Polar filament with 5 turns. Sporoplasm in posterior part of spore, bi-nucleated.

***Plasmodium***. Plasmodia polymorphous (round, spherical or elongated), often with finger-like processes averaging 20 μm in length (Fig. 3a). Plasmodia di- to polysporous, without visible pansporoblast formation, measuring 25–71 × 18–53 μm.

### Remarks

Five *Hoferellus* spp. have been found in nine clupeid species worldwide; of these four were reported from members of the genus *Alosa* but none of them from the allis shad (Table 1). *Hoferellus donecii* (Gasimagomedov, 1970) [30] and *H. jurachni* Moshu & Trombitsky, 2006 [31] differ considerably from *H. alosae* n. sp. with regard to spore shape and length as well as organization of the posterior spore appendages. *Hoferellus caspialosum* (Dogiel & Bychovsky 1939) [32] is smaller in size than *H. alosae* n. sp. *Hoferellus caudatus* (Parisi, 1910) [33] overlaps with *H. alosae* n. sp. with regard to most measurements. However, in contrast to *H. alosae* n. sp., this species was shown to consistently possess long posterior filaments, while *H. alosae* n. sp. was only occasionally found to have posterior filaments. Furthermore, the posterior end of the spore of *H. caudatus* is serrated whereas that of *H. alosae* n. sp. is smooth with only one small process on each valve. *Hoferellus caudatus* was described from an isolated, landlocked population of twaite shad *Alosa agone* (Scopoli, 1786) in Lake Como in Italy [33, 34], but was later reported from the anchovy *Engraulis encrasicolus* (L.) [35] and its Azov Sea-inhabiting subspecies *E. encrasicolus maeoticus* Pusanov, 1926 [36]. Considering the important difference in host habitat and the recently described strong host specificity of *Hoferellus* spp. in closely related cyprinids [12], it may be speculated that the records of Reshetnikova [35] and Naidenova [36] refer to a different parasite species in the anchovies. One could suspect a similar case for *H. caspialosum* which was described from the Caspian shad *Alosa caspia caspia* (Eichwald, 1838) by Dogiel & Bychovsky [32] but later reported from the Pontic shad *Alosa immaculata* Bennet, 1835 and the twait shad *Alosa fallax* (Lacépède, 1803) [37]. However, in contrast to *A. alosa*, the latter *Alosa* spp. are more closely related and their exact relationship is unresolved [38]. Unfortunately, SSU rDNA sequences are presently only available for *H. alosae* n. sp. from the allis shad.

**Table 1:** Summary of *Hoferellus* spp. reports from *Alosa* spp. including information on localities and morphological characteristics

				Plasmodia		Spores					
Species	Host records	Localities	Site of infection	Size (μm)	Description	Spore size (μm)	Description	Valve striations	Posterior processes	PC size (μm)	PC description
*H. alosae* n. sp.	*Alosa alosa* (L.)	Dordogne and Garonne (France), Rhine (Germany)	Renal tubules, ureters, urinary bladder	25–71 × 18–53	Polymorphous in shape, di- to polysporous, without pan-sporoblasts; surface with finger-like processes	L: 9.1–10.3 (9.7 ± 0.4); W: 7.7–9.2 (8.4 ± 0.5); T: 7.2–8.3 (7.7 ± 0.3)	Subspherical, pronounced suture line; single-celled bi-nucleated sporoplasm	12 longitudinal ridges	2, small, occasionally up to 7 hair-like filaments up to 22 μm long	L: 3.5–4.4 (4.0 ± 0.2); W: 2.4–3.6 (3.0 ± 0.3)	Equal in size, subspherical, pyriform; filament in 5 coils
*H. jurachni* Moshu & Trombitsky, 2006	*Alosa tanaica* (Grimm, 1901) [31]	Sasyk Lake, Cuciurgan reservoir	Renal tubules, ureters, urinary bladder	24–63 × 15–25, disporous pansporoblasts 12.5–16.5	Polymorphous in shape, polysporous, with disporous pansporoblasts; surface with small lobo- podia	L: 8.5–12.5 W: 6.4–7.5 T: 7.5–10.0	Triangular shape; flattened anterior pole, narrow posterior pole	4–8 longitudinal lines	Numerous short, lamellate processes surrounded by transparent mucous envelope	L: 3.5–4.0	Equal in size, spherical, pointed towards opening
*H. caudatus* (Parisi, 1910) (syns *Sphaerospora caudata*; *Mitraspora caudata*)	*Alosa agone* (Scopoli, 1786) [33]^a^;	Lake Como, Italy	Renal tubules		Polymorphous in shape, with disporous pansporoblasts and lobopodia	L: 10–11	Subspherical, valves thick (2–3 μm), pronounced suture line		Posterior end of valves serrated with 6 long filaments emerging from small projections	L: 4.0–4.5	
	*Engraulis encrasicolus* * maeoticus* (Pusanov, 1926) [35, 36]	Black Sea									
*H. caspialosum* (Dogiel & Bychovsky, 1939) (syn. *Sphaerospora caspialosae*)	*Alosa caspia caspia* (Eichwald, 1838) [32]^b^	Peninsula Sara, Caspian Sea, Azerbaijan	Renal tubules	12–15	Round in shape	L: 8.5; W: 7.7	Round to oval		Posterior end of spore with a number of small protrusions/projections; long filaments not observed		Polar filament in 4 coils
	*Alosa immaculata* Bennet, 1835^c^; *Alosa fallax*, (Lacépède, 1803) [37]^d^	Black Sea									

*Abbreviations*: *L* length, *W* width, *T* thickness, *PC* polar capsule

^a^Reported as *A. finta lacustris* (Fatio, 1890)

^b^report is related to jun. syn. *Caspialosa caspia* (Eichwald, 1838)

^c^report is related to jun. syn. *Alosa kessleri pontica* (Eichwald, 1838)

^d^report is related to *Alosa fallax nilotica* (Geoffroy Saint-Hilaire, 1809)

### Pathology and diagnostics

The gross examination of allis shad revealed no pathological or macroscopically visible changes of the kidney. In allis shad from the Garonne/Dordogne system the intensity of infection with *Hoferellus alosae* n. sp. was estimated as mild in 45 %, moderate in 47 % and severe in 9 % of cases, while the infection intensity of allis shad from the Rhine was always mild. Depending on the intensity of infection, the tubules were mildly to severely dilated, but generally no further pathological changes of the kidney tubules, the parenchyma or the excretory ducts were observed. In a single severe case the rupture of dilated renal tubules led to an inflow of *H. alosae* n. sp. in the surrounding kidney parenchyma causing an inflammation with infiltration of epitheloid cells.

The PCR assay designed in this study was specific for *H. alosae* n. sp. and did not amplify DNA of any phylogenetically related species screened in this study (see Methods).

### SSU rDNA sequence diversity in host individuals and river systems

In allis shad from the Dordogne/Garonne river system, all sequences obtained by direct sequencing of PCR products and by sequencing of clones belonged to a single species, *H. alosae* n. sp. In contrast, in shad from the Rhine, *H. alosae* n. sp. was amplified from two individuals that had mixed infections of *H. alosae* n. sp. with a second myxozoan inhabiting the urinary tract of allis shad. This second myxozoan was later morphologically identified as *Ortholinea* sp., via spores detected in histological sections and the partial SSU rDNA sequence was submitted to GenBank under the accession number KU301053. *Ortholinea* sp. was also detected in a third fish from the Rhine (identified by SSU rDNA sequences from the nested myxozoan PCR assay), which had no mixed infection with *H. alosae* n. sp.

Almost full length SSU rDNA PCR products of *H. alosae* n. sp. in numerous fish from the Garonne did not exhibit variable positions or polymorphic sites, whereas those in allis shad from the Dordogne showed six consistent single nucleotide polymorphisms (SNPs) at positions 100 (A/G), 524 (C/T), 527 (C/T), 590 (A/G), 608 (C/T), 825 (C/T), in a 1923 bp alignment. The comparison of cloned partial SSU rDNA sequences (901 bp) for *H. alosae* n. sp. encompassing these sites, revealed two additional polymorphic sites (total of 8) and showed that clones from individual fish varied in only 3–7 base changes (1 SNP) in the Garonne, in 9–10 base changes (6 SNPs) in the Dordogne and in 15–18 base changes (2 SNPs) in the Rhine (summarized in Table 2; detailed in Additional files 2, 3 and 4: Tables S2, Tables S3 and Tables S4, see alignment in Additional file 5: Figure S1). Only a single SNP site overlapped between different rivers (position 655, rivers Rhine and Garonne). The genetic diversity comparison between young-of-the-year shad and adult fish returning to spawn (Garonne) showed a similar signature, with 3–7 base changes *vs* 3–5 base changes and the presence of a single, identical SNP (Table 2, Additional file 5: Figure S1). Sequence divergence between complete SSU rDNA sequences (PCR products of 19 fish; 1922 bp) was 0–1.09 % and that between partial cloned SSU rDNA isolates (66 sequences; 901 bp) was 0–1.44 %.

**Table 2:** *Hoferellus alosae* n. sp. SSU rDNA diversity in different rivers. Location and frequency of single nucleotide polymorphic (SNP) site changes and number of individual nucleotide changes identified in 901 bp cloned SSU rDNA fragments of *H. alosae* n. sp. from *Alosa alosa* (6 clones per fish sequenced)

River-fish individual^a^	SNP sites and number of changes								Other nt changes	Total sites with nt changes
	91 C → T	95 A → G	519 T → C	522 T → C	585 A → G	603 C → T	655 C → T	820 C → T		
Garonne - F39	–	–	–	–	–	–	1/6	–	4	5
Garonne - F240	–	–	–	–	–	–	1/6	–	2	3
Garonne - F243	–	–	–	–	–	–	1/6	1/6^b^	3	5
Garonne - F241	–	–	–	–	–	–	1/6	–	6	7
Garonne - F247	–	–	–	–	–	–	–	–	3	3
Garonne - F248	–	–	–	–	–	–	2/6	–	4	5
Dordogne - F179	–	6/6	6/6	6/6	6/6	6/6	–	6/6	4	10
Dordogne - F187	–	1/6	1/6	1/6	1/6	1/6	–	2/6	3	9
Dordogne - F188	–	3/6	5/6	5/6	2/6	5/6	–	5/6	3	9
Rhine - D281	3/6	–	–	–	–	–	–	–	14	15
Rhine - D290	1/6	–	–	–	–	–	6/6	–	16	18

^a^Compare Additional file 1: Table S1

^b^Not considered a polymorphic site in Garonne as change observed in only 1/36 clones

### Phylogenetic relationships

**Fig. 4:**
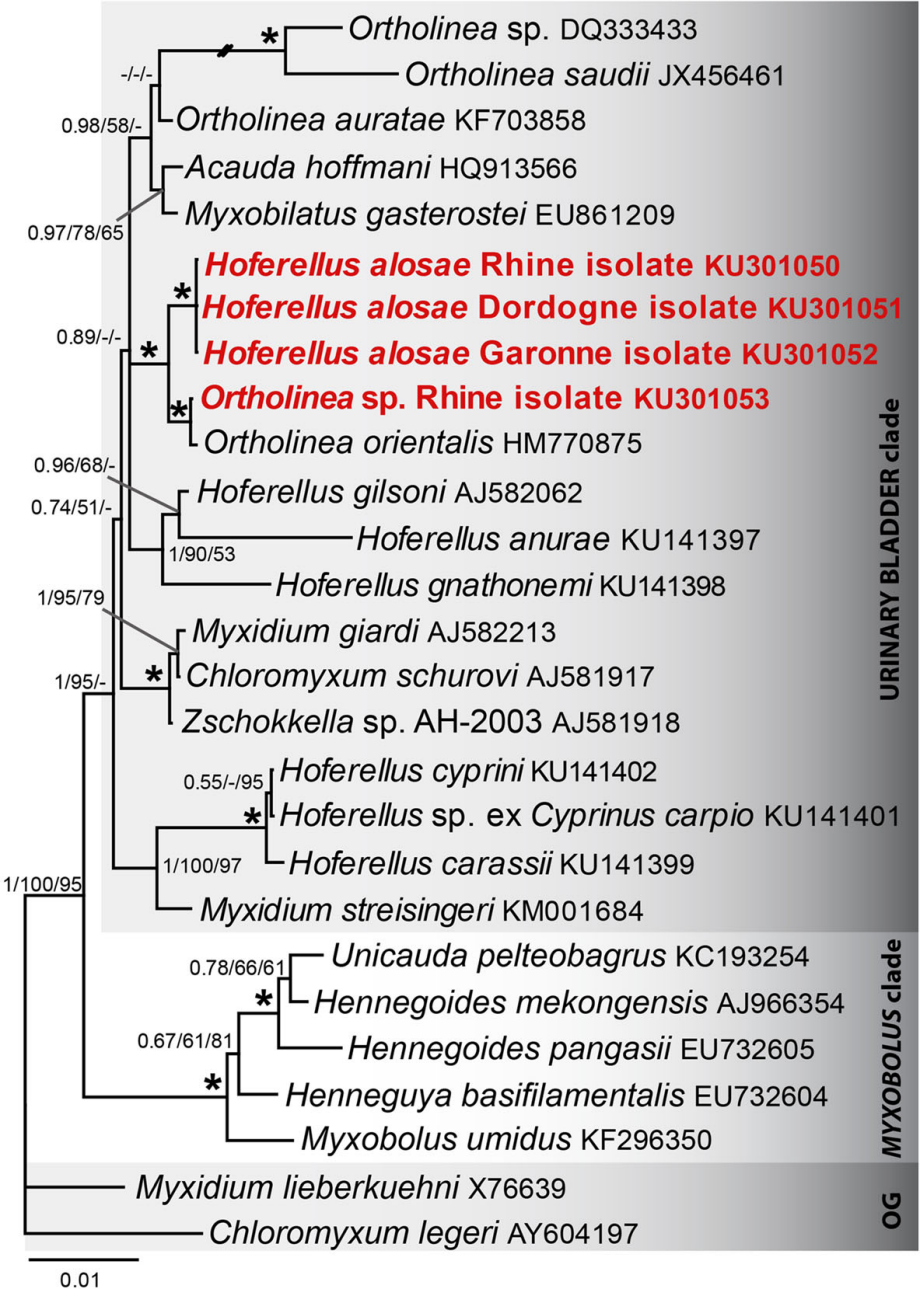
Bayesian inference (BI) tree showing the phylogenetic position of *Hoferellus alosae* n. sp. and *Ortholinea* sp. within the freshwater urinary bladder clade as defined by Fiala [25]. The new sequences are shown in *bold* and *red. Myxidium lieberkuehni* and *Chloromyxum legeri* were used as outgroup (OG). The short diagonal double-line represents a branch shortened to 50 % of its original length. Dashes at nodes represent nodal supports MP/ML < 50 and BI < 0.60 or node not present in the maximum parsimony (MP) or Maximum likelihood (ML) tree. Asterisk labels a node with maximum nodal supports (MP/ML = 100; BI = 1)

BLAST results and subsequent pairwise sequence alignments indicated *O. orientalis* as the closest relative of *H. alosae* n. sp., with only 87–88 % SSU rDNA sequence identity. The second myxozoan SSU rDNA sequence belongs to *Ortholinea* sp. and was isolated from three fish in the Rhine. The sequence was almost identical to *O. orientalis*, with only 1.8–2.0 % sequence divergence over 912 bp. A consistent number of 15 nucleotide changes in the variable regions of the SSU rDNA suggest *Ortholinea* sp. is a different, but very closely related species. Phylogenetic analyses (MP/ML/BI; Fig. 4) showed that *Ortholinea* sp. clusters with *O. orientalis* and the three *H. alosae* n. sp. river isolates in a well-supported group (Fig. 4). This group furthermore clustered in polytomy with two clades composed of three *Hoferellus* (*sensu lato*) spp. (i.e. *H. gilsoni* (Debaisieux, 1925), *H. anurae* and *H. gnathonemi*) and of *Ortholinea* spp. + *Myxobilatus gasterostei* (Parisi, 1912) + *Acauda hoffmani* Whipps, 2011. Importantly, *H. alosae* n. sp. clustered outside the *Hoferellus* (*sensu stricto*) clade (comprising the type-species *H. cyprini* as well as *H. carassii* and *Hoferellus* sp. ex *Cyprinus carpio* L.) and hence is to be considered *Hoferellus* (*sensu lato*).

## Discussion

Little is known about the population structure and dynamics of myxozoans, and information on the *de novo* establishment of myxozoans in watersheds that have been extirpated of obligatory fish host populations is missing to date. The repatriation program of the allis shad, *A. alosa*, in the Rhine allowed a unique first insight into the repopulation by myxozoans, and the diversity of new parasite settlements compared with watersheds where hosts and parasites have coexisted for a long period of time.

Baglinière & Élie [39] listed 16 species of *Alosa* native to the northern hemisphere and distributed through the western and eastern Atlantic coasts, the Mediterranean, Black and Caspian Seas, as well as Lake Volvi (Greece). The genus shows a large variation in life-history strategies (mostly anadromous, but also amphidromous, entirely marine and strictly freshwater) and a capacity to colonize new habitats thus making the genus *Alosa* an interesting model to study speciation and adaptation of the host itself and its parasites. To date, four *Hoferellus* spp. have been described from the urinary tract of *Alosa* spp. (Table 1). However, due to the different life strategies and hence geographical isolation (see also remarks in the species description) and strong host specificity in coelozoic myxozoans (e.g. [40–42]) with limited but convincing evidence also from the genus *Hoferellus* [14], parasite diversity in the genus *Alosa* may be larger than presently estimated. Unfortunately, sequence data are only available for *H. alosae* n. sp. from *A. alosa* (present study). Phylogenetic analyses of SSU rDNA sequences for *Hoferellus* spp. from different *Alosa* spp. and geographical localities in relation to host phylogeny would shed light on the diversity of species and the co-evolutionary history of *Alosa* spp. and their *Hoferellus* spp. parasites.

In myxozoans, species boundaries are difficult to determine as fish are unlikely infected by only one spore from a single parasite clone produced in one invertebrate host individual. SSU rDNA sequences have been widely used to describe and diagnose myxozoan species. rDNA occurs in a number of copies in eukaryotic cells [43], with around estimated 1000 copies in myxozoan rDNA [44–46]. Despite the concerted evolution of the rRNA gene [47], some degree of variation exists between these copies. Hence, rDNA sequences obtained from a single fish host show the full spectrum of such intragenomic heterogeneity as well as of intraspecific heterogeneity between ‘strains’ or genotypes. When assuming intragenomic heterogeneity as a constant in isolates of a single species, any additional variation can be ascribed to host- or site-specific variation. Comprehensive data on intraspecific variation of rDNA sequences are available for only a few myxozoan species, *Myxobolus cerebralis* Hofer, 1903 [48, 49], *Tetracapsuloides bryosalmonae* Canning, Curry, Feist, Longshaw & Okamura, 1999 [47, 48], *Kudoa thyrsites* (Gilchrist, 1924) [50], *Ceratonova shasta* (Noble, 1950) [51, 52] and *Parvicapsula minibicornis* Kent, Whitaker & Dawe, 1977 [53]. In most cases, variations have been ascribed to geographical differences between isolates. However, in the case of *C. shasta,* four sympatric genotypes were described, that showed little geographical structure in the parasite population but profound population isolation effects created by utilizing different vertebrate hosts. To some extent, population structuring by fish host was also evident in coho and Chinook salmon in *P. minibicornis*. In contrast to these species, *H. alosae* n. sp. seems to be host-specific and geographical isolation appears to be the main factor for SSU rDNA site variability.

Reports of allis shad populations in the Garonne and the Dordogne date back to the end of the 18th Century [54] and stocks are well-established despite a present decline [55]. *Hoferellus alosae* n. sp. SSU rDNA sequence variation is larger in hosts from the Dordogne (9–10 nucleotide positions and 6 SNPs) than in the Garonne (3–7 nucleotide positions, 1 SNP), potentially indicating a higher diversity in hosts from the Dordogne. This is surprising since the Garonne is longer (575 *vs* 472 km) and has a much larger number of tributaries, suggesting a higher diversity of invertebrate host habitats and populations of *H. alosae* n. sp. [56]. However, the distribution of susceptible oligochaete species is correlated to a variety of conditions [51, 57–59] and remains poorly understood. Independently, the presence of six SNP positions out of a total of 9–10 nucleotide changes indicates the establishment of a locally different ‘strain’ or genotype of *H. alosae* n. sp. in the Dordogne watershed when compared with that of the Garonne. *Ceratonova shasta* only showed three SNP SSU rDNA sites while *P. minibicornis* exhibited 17 SNPs [51, 53]. In contrast to these two species, our study was limited to a comparatively small sample set from three river systems (Garonne/Dordogne and Rhine) that revealed eight SNPs. The population dynamics of *C. shasta* and *P. minibicornis* are considerably different as a number of genotypes exist in different salmonid hosts and a polychaete definitive host is used in both cases [52, 53]. *Hoferellus alosae* n. sp. is expected to be very host-specific and to use an oligochaete definitive host, since it clusters in the ‘freshwater’ clade of myxozoans as defined by Fiala [25] whose members parasitize oligochaetes [60]. We expected to find more overlap in SNPs between the geographically close and estuary-linked French rivers. In contrast, our present data indicate a clear separation of *H. alosae* n. sp. populations in these rivers, which are characterized by a unique nucleotide signature for each river. The overlapping nucleotide signature pattern in young-of-the-year and adults in the Garonne indicate that straying of allis shad is limited, resulting in the establishment of local successful parasite genotypes as a consequence of long-term co-evolution of *H. alosae* n. sp. and allis shad in a specific watershed or microhabitat.

The SSU rDNA sequence variability pattern of *H. alosae* n. sp. in the first returners (2014) of allis shad repatriated in the Rhine was defined by only one (out of two) common SNP. The large number of individual changes in different clones (14–16) may indicate the existence of further SNP sites which cannot be identified at present. Likely, they represent those of close-by rivers, such as e.g. the Scheldt or the Meuse, where allis shad may be straying to or from. The large number of changes is likely a result of geographical distance from the French rivers. However, the nucleotide signatures (known SNPs and single nucleotide changes) vary to the same extent between the isolates from the Rhine as between Rhine and Garonne or Dordogne, rivers in which SNP signatures are site-specific and relatively homogenous. This strongly indicates that the infection in the two Rhine shad originates from at least two different sources and that genetic diversity in repatriated allis shad in the Rhine is higher than that found in established fish populations in the French rivers. Since the majority of the changes represents single nucleotide changes it may even be speculated that they represent multi-site signatures. The recovered isolates show a complexity that requires further analysis. In order to determine the diversity of *H. alosae* n. sp. isolates and populations in different watersheds as well as riverine microhabitats in more depth, the analysis of polymorphic microsatellite loci would be desirable as it allows a better fine scaling of the population structure in complex organisms like myxozoans that are characterized by clonal, vegetative and sexual reproduction. However, our findings underline that the analysis of parasites and their genetic diversity is a valuable tool to investigate biological characteristics and ecosystem variations.

The twaite shad, *A. fallax* shares *A. alosa*’s history of population decrease in the middle of the 20th Century but small populations can now be found restricted to the lower Rhine [61]. Though unlikely identical with *H. caspialosum* (originally described from geographically isolated *A. caspia caspia*, see Table 1 and Remarks section), the twaite shad in the Rhine may still harbor *Hoferellus* infection. In phylogenetic studies, *A. alosa* and *A. fallax* form clearly distinct lineages [38], even in watersheds where they hybridize [62]. The question arises whether the twaite shad can serve as a reservoir host for *H. alosae* n. sp. in the Rhine. Arguments that reject this hypothesis and the consequential existence of *H. alosae* n. sp.-infected oligochaete populations in the Rhine are: (i) Low infection rates of *A. alosa* [adults 22.2 % (2/9), young-of-the-year 0 % (0/26)] in contrast to the French rivers where 100 % of adults and 63.6 % of juveniles harbor infection, confirming the high prevalences typical for myxozoans in riverine habitats with effective dissemination of infective spore stages in the water current (e.g. [63]), and (ii) The high genetic diversity and lack of an identical genetic signature of *H. alosae* n. sp. isolates from the Rhine. *Alosa fallax* were not examined in the course of this project, however, these circumstances strongly suggest that *A. fallax* does not serve as a reservoir host for *H. alosae* n. sp. More likely, infection in the returning adults results from their contact with different *H. alosae* n. sp. enzootic watersheds. Alternatively, oligochaetes in parts of the river may become infected by spores released from straying allis shad originating from elsewhere or by spores released via the faeces of migratory piscivorous birds. The importance of avian vectors in the translocation of spores is unexplored to date but *M. cerebralis* spores are able to infect oligochaetes after avian intestinal passage and are released for several days after fish consumption [64]. New invasion and colonization events are generally associated with founder effects that reduce genetic variation in incipient populations [65]. However, due to the abundance and diversity of myxozoans in different aquatic habitats and a variety of source localities for *de novo* invasion this appears to be the opposite in this group. High SSU rDNA diversity in *H. alosae* n. sp. from the Rhine and the presence of another myxozoan, *Ortholinea* sp., which was absent from the French watersheds but present in three out of nine adult shad in the Rhine, suggests that myxozoan parasite introduction into parasite-free territory is characterized by a high diversity of species and ‘strains’. To our knowledge, this is the first time that initial parasite introduction data are available for myxozoans. Monitoring of the infections in repatriated allis shad returning to spawn over following years will allow to obtain real-time data on within-host competition and the survival and predominant establishment of the fittest in the Rhine.

## Conclusion

To the best of our knowledge, this is the first study on a *de novo* introduction of a myxozoan species in a river where the host fish population was repatriated after 70 years of absence and loss of infection from the alternate invertebrate host. The first adults of allis shad returning to spawn in the Rhine showed low infection prevalence of *H. alosae* n. sp. when compared to established fish populations in France, where all adults were found infected. However, the diversity of *H. alosae* n. sp. clones in only two infected hosts varied as strongly between the Rhine isolates as between the Rhine and two rivers with established fish populations and *H. alosae* n. sp. infections in France. Additionally, a second species, *Ortholinea* sp., was only found in the Rhine. This comparatively high diversity in the newly established host population can only be explained by the introduction of spores from genetically diverse sources, most likely via straying hosts. The allis shad repatriation study in the Rhine presently offers the unique opportunity for a long-term study of a *de novo* settlement of this host-specific myxozoan and its succession from high diversity as a result of multiple introductions to a locally established, presumably successful strain, which characterize each of the French rivers.

## Additional files

Additional file 1: Table S1. Summary of samples obtained from *Alosa alosa*. (DOCX 48 kb) Additional file 2: Table S2. SSU rDNA variability of *Hoferellus alosae* n. sp. clones from fish individuals in the Garonne. (DOCX 18 kb) Additional file 3: Table S3. SSU rDNA variability of *Hoferellus alosae* n. sp. clones from fish individuals in the Dordogne. (DOCX 17 kb) Additional file 4: Table S4. SSU rDNA variability of *Hoferellus alosae* n. sp. clones from fish individuals in the Rhine. (DOCX 16 kb) Additional file 5: Figure S1. Alignment of cloned SSU rDNA fragments of *Hoferellus alosae* n. sp. (901 bp) obtained from different fish individuals and river systems (see Additional file 1: Table S1 for origin and characteristics of samples). Single nucleotide polymorphic sites (SNPs) and individual nucleotide changes marked in color. (PNG 557 kb)

## Abbreviations

BI: Bayesian inference
EU-LIFE: European Union -L’Instrument Financier pour l’Environnement
MP: Maximum parsimony
ML: Maximum likelihood
SNP: Single nucleotide polymorphism
SSU rDNA: Small subunit ribosomal deoxyribonucleic acid
