# Supplementary material for: Repatriation of an old fish host as an opportunity for myxozoan parasite diversity: The example of the allis shad, Alosa alosa (Clupeidae), in the Rhine
Source: Parasit Vectors. 2016 Sep 15;9:505. doi: 10.1186/s13071-016-1760-6 (PMC5024467; doi:10.1186/s13071-016-1760-6)
Supplement: Additional file 1: Table S1. — Summary of samples obtained from Alosa alosa. (DOCX 48 kb) [file 13071_2016_1760_MOESM1_ESM.docx]

**Additional file 2: Table S1.** Summary of samples (YOY = young-of-the-year, K = kidney, W = whole fish, F = frozen, E = 100% ethanol, H= 5 % formalin, PCR=sequences from PCR product, CL=sequences from clones, nd = no data)

| **Origin of material** | | | | | | **Examination** | | | | | |
| --- | --- | --- | --- | --- | --- | --- | --- | --- | --- | --- | --- |
| **Year of sampling** | **Sample No.** | **Country** | **River** | **Coordinates** | **Age** | **Sample** | **nativ** | **Mor-phology** | **Histo-logy-result** | **PCR-result** | **Sequencing** |
| 2012 | F 1 | France | Garonne | Golfech (44° 06′ 33“ N, 0° 51′ 14′′ O) | adult | K: F, H | nd | nd | + | + | nd |
| 2012 | F 3 | France | Garonne | Golfech (44° 06′ 33“ N, 0° 51′ 14′′ O) | adult | K: F, H | nd | nd | + | + | nd |
| 2012 | F 4 | France | Garonne | Golfech (44° 06′ 33“ N, 0° 51′ 14′′ O) | adult | K: H | nd | nd | + | nd | nd |
| 2012 | F 5 | France | Garonne | Golfech (44° 06′ 33“ N, 0° 51′ 14′′ O) | adult | K: H | nd | nd | + | nd | nd |
| 2012 | F 6 | France | Garonne | Golfech (44° 06′ 33“ N, 0° 51′ 14′′ O) | adult | K: F, H | nd | nd | + | + | nd |
| 2012 | F 10 | France | Garonne | Golfech (44° 06′ 33“ N, 0° 51′ 14′′ O) | adult | K: H | nd | nd | + | nd | nd |
| 2012 | F 12 | France | Garonne | Golfech (44° 06′ 33“ N, 0° 51′ 14′′ O) | adult | K: H | nd | nd | + | nd | nd |
| 2012 | F 13 | France | Garonne | Golfech (44° 06′ 33“ N, 0° 51′ 14′′ O) | adult | K: H | nd | nd | + | nd | nd |
| 2012 | F 15 | France | Garonne | Golfech (44° 06′ 33“ N, 0° 51′ 14′′ O) | adult | K: H | nd | nd | + | nd | nd |
| 2012 | F 17 | France | Garonne | Golfech (44° 06′ 33“ N, 0° 51′ 14′′ O) | adult | K: H | nd | nd | + | nd | nd |
| 2012 | F 18 | France | Garonne | Golfech (44° 06′ 33“ N, 0° 51′ 14′′ O) | adult | K: H | nd | nd | + | nd | nd |
| 2012 | F 19 | France | Garonne | Golfech (44° 06′ 33“ N, 0° 51′ 14′′ O) | adult | K: H | nd | nd | + | nd | nd |
| 2012 | F 20 | France | Garonne | Golfech (44° 06′ 33“ N, 0° 51′ 14′′ O) | adult | K: H | nd | nd | + | nd | nd |
| 2012 | F 22 | France | Garonne | Golfech (44° 06′ 33“ N, 0° 51′ 14′′ O) | adult | K: H | nd | nd | + | nd | nd |
| 2012 | F 25 | France | Garonne | Golfech (44° 06′ 33“ N, 0° 51′ 14′′ O) | adult | K: H | nd | nd | + | nd | nd |
| 2012 | F 26 | France | Garonne | Golfech (44° 06′ 33“ N, 0° 51′ 14′′ O) | adult | K: H | nd | nd | + | nd | nd |
| 2012 | F 27 | France | Garonne | Golfech (44° 06′ 33“ N, 0° 51′ 14′′ O) | adult | K: H | nd | nd | + | nd | nd |
| 2012 | F 28 | France | Garonne | Golfech (44° 06′ 33“ N, 0° 51′ 14′′ O) | adult | K: H | nd | nd | + | nd | nd |
| 2012 | F 29 | France | Garonne | Golfech (44° 06′ 33“ N, 0° 51′ 14′′ O) | adult | K: H | nd | nd | + | nd | nd |
| 2012 | F 30 | France | Garonne | Golfech (44° 06′ 33“ N, 0° 51′ 14′′ O) | adult | K: H | nd | nd | + | nd | nd |
| 2012 | F 31 | France | Garonne | Golfech (44° 06′ 33“ N, 0° 51′ 14′′ O) | adult | K: H | nd | nd | + | nd | nd |
| 2012 | F32 | France | Garonne | Golfech (44° 06′ 33“ N, 0° 51′ 14′′ O) | adult | K: H | nd | nd | + | nd | nd |
| 2012 | F 33 | France | Garonne | Golfech (44° 06′ 33“ N, 0° 51′ 14′′ O) | adult | K: H | nd | nd | + | nd | nd |
| 2012 | F 34 | France | Garonne | Golfech (44° 06′ 33“ N, 0° 51′ 14′′ O) | adult | K: H | nd | nd | + | nd | nd |
| 2012 | F 36 | France | Garonne | Golfech (44° 06′ 33“ N, 0° 51′ 14′′ O) | adult | K: H | nd | nd | + | nd | nd |
| 2012 | F 37 | France | Garonne | Golfech (44° 06′ 33“ N, 0° 51′ 14′′ O) | adult | K: H | nd | nd | + | nd | nd |
| 2012 | F 38 | France | Garonne | Golfech (44° 06′ 33“ N, 0° 51′ 14′′ O) | adult | K: H | nd | nd | + | nd | nd |
| 2012 | F 39 | France | Garonne | Golfech (44° 06′ 33“ N, 0° 51′ 14′′ O) | adult | K: F, H | nd | nd | + | + | PCR/CL |
| 2012 | F 40 | France | Garonne | Golfech (44° 06′ 33“ N, 0° 51′ 14′′ O) | adult | K: H | nd | nd | + | nd | nd |
| 2012 | F 41 | France | Garonne | Golfech (44° 06′ 33“ N, 0° 51′ 14′′ O) | adult | K: H | nd | nd | + | nd | nd |
| 2012 | F 42 | France | Garonne | Golfech (44° 06′ 33“ N, 0° 51′ 14′′ O) | adult | K: H | nd | nd | + | nd | nd |
| 2013 | F 134 | France | Garonne | Golfech (44° 06′ 33“ N, 0° 51′ 14′′ O) | adult | K: F, H | - | nd | + | + | nd |
| 2013 | F 135 | France | Garonne | Golfech (44° 06′ 33“ N, 0° 51′ 14′′ O) | adult | K: H | - | nd | + | nd | nd |
| 2013 | F 136 | France | Garonne | Golfech (44° 06′ 33“ N, 0° 51′ 14′′ O) | adult | K: H | - | nd | - | nd | nd |
| 2013 | F 137 | France | Garonne | Golfech (44° 06′ 33“ N, 0° 51′ 14′′ O) | adult | K: H | + | nd | + | nd | nd |
| 2013 | F 138 | France | Garonne | Golfech (44° 06′ 33“ N, 0° 51′ 14′′ O) | adult | K: F, H | + | nd | + | + | nd |
| 2013 | F 139 | France | Garonne | Golfech (44° 06′ 33“ N, 0° 51′ 14′′ O) | adult | K: H | + | nd | + | nd | nd |
| 2013 | F 140 | France | Garonne | Golfech (44° 06′ 33“ N, 0° 51′ 14′′ O) | adult | K: H | + | nd | + | nd | nd |
| 2013 | F 141 | France | Garonne | Golfech (44° 06′ 33“ N, 0° 51′ 14′′ O) | adult | K: H | + | nd | + | nd | nd |
| 2013 | F 142 | France | Garonne | Golfech (44° 06′ 33“ N, 0° 51′ 14′′ O) | adult | K: H | + | nd | + | nd | nd |
| 2013 | F 143 | France | Garonne | Golfech (44° 06′ 33“ N, 0° 51′ 14′′ O) | adult | K: H | - | nd | + | nd | nd |
| 2013 | F 144 | France | Garonne | Golfech (44° 06′ 33“ N, 0° 51′ 14′′ O) | adult | K: H | + | nd | + | nd | nd |
| 2013 | F 145 | France | Garonne | Golfech (44° 06′ 33“ N, 0° 51′ 14′′ O) | adult | K: H | + | nd | + | nd | nd |
| 2013 | F 146 | France | Garonne | Golfech (44° 06′ 33“ N, 0° 51′ 14′′ O) | adult | K: H | + | nd | + | nd | nd |
| 2013 | F 147 | France | Garonne | Golfech (44° 06′ 33“ N, 0° 51′ 14′′ O) | adult | K: H | + | nd | + | nd | nd |
| 2013 | F 148 | France | Garonne | Golfech (44° 06′ 33“ N, 0° 51′ 14′′ O) | adult | K: H | + | nd | + | nd | nd |
| 2013 | F 149 | France | Garonne | Golfech (44° 06′ 33“ N, 0° 51′ 14′′ O) | adult | K: H | + | nd | + | nd | nd |
| 2013 | F 150 | France | Garonne | Golfech (44° 06′ 33“ N, 0° 51′ 14′′ O) | adult | K: H | + | nd | + | nd | nd |
| 2013 | F 151 | France | Garonne | Golfech (44° 06′ 33“ N, 0° 51′ 14′′ O) | adult | K: H | + | nd | + | nd | nd |
| 2013 | F 152 | France | Garonne | Golfech (44° 06′ 33“ N, 0° 51′ 14′′ O) | adult | K: H | + | nd | + | nd | nd |
| 2013 | F 153 | France | Garonne | Golfech (44° 06′ 33“ N, 0° 51′ 14′′ O) | adult | K: H | + | nd | + | nd | nd |
| 2013 | F 154 | France | Garonne | Golfech (44° 06′ 33“ N, 0° 51′ 14′′ O) | adult | K: H | + | nd | + | nd | nd |
| 2013 | F 155 | France | Garonne | Golfech (44° 06′ 33“ N, 0° 51′ 14′′ O) | adult | K: H | + | nd | + | nd | nd |
| 2013 | F 156 | France | Garonne | Golfech (44° 06′ 33“ N, 0° 51′ 14′′ O) | adult | K: H | + | nd | + | nd | nd |
| 2013 | F 157 | France | Garonne | Golfech (44° 06′ 33“ N, 0° 51′ 14′′ O) | adult | K: H | + | nd | + | nd | nd |
| 2013 | F 158 | France | Garonne | Golfech (44° 06′ 33“ N, 0° 51′ 14′′ O) | adult | K: H | + | nd | + | nd | nd |
| 2013 | F 159 | France | Garonne | Golfech (44° 06′ 33“ N, 0° 51′ 14′′ O) | adult | K: H | + | nd | + | nd | nd |
| 2013 | F 160 | France | Garonne | Golfech (44° 06′ 33“ N, 0° 51′ 14′′ O) | adult | K: H | + | nd | + | nd | nd |
| 2013 | F 161 | France | Garonne | Golfech (44° 06′ 33“ N, 0° 51′ 14′′ O) | adult | K: F, H | + | nd | + | + | nd |
| 2013 | F 162 | France | Garonne | Golfech (44° 06′ 33“ N, 0° 51′ 14′′ O) | adult | K: F, H | + | nd | + | + | nd |
| 2013 | F 163 | France | Dordogne | Tuillière (44° 50′ 42″ N 0° 37′ 59″ E) | adult | K: H | - | nd | - | nd | nd |
| 2013 | F 164 | France | Dordogne | Tuillière (44° 50′ 42″ N 0° 37′ 59″ E) | adult | K: H | - | nd | - | nd | nd |
| 2013 | F 165 | France | Dordogne | Tuillière (44° 50′ 42″ N 0° 37′ 59″ E) | adult | K: H | - | nd | - | nd | nd |
| 2013 | F 166 | France | Dordogne | Tuillière (44° 50′ 42″ N 0° 37′ 59″ E) | adult | K: H | + | nd | + | nd | nd |
| 2013 | F 167 | France | Dordogne | Tuillière (44° 50′ 42″ N 0° 37′ 59″ E) | adult | K: H | + | nd | + | nd | nd |
| 2013 | F 168 | France | Dordogne | Tuillière (44° 50′ 42″ N 0° 37′ 59″ E) | adult | K: H | + | nd | + | nd | nd |
| 2013 | F 169 | France | Dordogne | Tuillière (44° 50′ 42″ N 0° 37′ 59″ E) | adult | K: H | + | nd | + | nd | nd |
| 2013 | F 170 | France | Dordogne | Tuillière (44° 50′ 42″ N 0° 37′ 59″ E) | adult | K: H | + | nd | + | nd | nd |
| 2013 | F 171 | France | Dordogne | Tuillière (44° 50′ 42″ N 0° 37′ 59″ E) | adult | K: H | + | nd | + | nd | nd |
| 2013 | F 172 | France | Dordogne | Tuillière (44° 50′ 42″ N 0° 37′ 59″ E) | adult | K: H | + | nd | + | nd | nd |
| 2013 | F 173 | France | Dordogne | Tuillière (44° 50′ 42″ N 0° 37′ 59″ E) | adult | K: H | + | nd | + | nd | nd |
| 2013 | F 174 | France | Dordogne | Tuillière (44° 50′ 42″ N 0° 37′ 59″ E) | adult | K: H | + | nd | + | nd | nd |
| 2013 | F 175 | France | Dordogne | Tuillière (44° 50′ 42″ N 0° 37′ 59″ E) | adult | K: H | + | nd | + | nd | nd |
| 2013 | F 176 | France | Dordogne | Tuillière (44° 50′ 42″ N 0° 37′ 59″ E) | adult | K: H | + | nd | + | nd | nd |
| 2013 | F 177 | France | Dordogne | Tuillière (44° 50′ 42″ N 0° 37′ 59″ E) | adult | K: H | + | nd | + | nd | nd |
| 2013 | F 178 | France | Dordogne | Tuillière (44° 50′ 42″ N 0° 37′ 59″ E) | adult | K: H | + | nd | + | nd | nd |
| 2013 | F 179 | France | Dordogne | Tuillière (44° 50′ 42″ N 0° 37′ 59″ E) | adult | K: E, H | + | nd | + | + | PCR/CL |
| 2013 | F 180 | France | Dordogne | Tuillière (44° 50′ 42″ N 0° 37′ 59″ E) | adult | K: E, H | + | nd | + | + | PCR |
| 2013 | F 181 | France | Dordogne | Tuillière (44° 50′ 42″ N 0° 37′ 59″ E) | adult | K: E, H | + | nd | + | + | PCR |
| 2013 | F 182 | France | Dordogne | Tuillière (44° 50′ 42″ N 0° 37′ 59″ E) | adult | K: E, H | + | nd | + | + | nd |
| 2013 | F 183 | France | Dordogne | Tuillière (44° 50′ 42″ N 0° 37′ 59″ E) | adult | K: E, H | + | nd | + | + | PCR |
| 2013 | F 184 | France | Dordogne | Tuillière (44° 50′ 42″ N 0° 37′ 59″ E) | adult | K: E, H | + | nd | + | + | PCR |
| 2013 | F 185 | France | Dordogne | Tuillière (44° 50′ 42″ N 0° 37′ 59″ E) | adult | K: E, H | + | nd | + | + | PCR |
| 2013 | F 186 | France | Dordogne | Tuillière (44° 50′ 42″ N 0° 37′ 59″ E) | adult | K: E, H | + | nd | + | + | PCR |
| 2013 | F 187 | France | Dordogne | Tuillière (44° 50′ 42″ N 0° 37′ 59″ E) | adult | K: E, H | + | nd | + | + | PCR/CL |
| 2013 | F 188 | France | Dordogne | Tuillière (44° 50′ 42″ N 0° 37′ 59″ E) | adult | K: E, H | + | nd | + | + | PCR/CL |
| 2013 | F 217 | France | Garonne | Couthures | YOY | W: B | nd | nd | - | nd | nd |
| 2013 | F 221 | France | Garonne | Couthures | YOY | K: E | nd | nd | nd | + | nd |
| 2013 | F 223 | France | Garonne | Couthures | YOY | K: E | nd | nd | nd | + | nd |
| 2013 | F 226 | France | Garonne | Couthures | YOY | K: E | nd | nd | nd | + | nd |
| 2013 | D 232 | Germany | Rhine | Landeshafen Wörth (49° 4' 4,4" N; 8°18' 34,5”E) | adult | K: E | - | nd | nd | - | nd |
| 2013 | D 264 | Germany | Rhine | Philippsburg (49° 4’ 9.3” N; 8° 25’ 55.2” E) | YOY | W: E | nd | nd | - | nd | nd |
| 2014 | D 233 | Germany | Rhine | Rees (51°45'18.00"N; 6°23'36.30"E) | adult | K: E, H | - | YES | - | - | nd |
| 2014 | D 234 | Germany | Rhine | Rees (51°45'18.00"N; 6°23'36.30"E) | adult | K: E, H | - | YES | - | - | nd |
| 2014 | D 235 | Germany | Rhine | Speyer (49°24'19.20"N; 8°29'40.25"E) | adult | K, F: E, H | - | nd | - | - | nd |
| 2014 | D 236 | Germany | Rhine | Speyer (49°24'19.20"N; 8°29'40.25"E) | adult | K, F: E, H | - | nd | - | - | nd |
| 2014 | F 238 | France | Garonne | Golfech (44° 06′ 33“ N, 0° 51′ 14′′ O) | adult | K: E, H | + | YES | + | + | nd |
| 2014 | F 239 | France | Garonne | Golfech (44° 06′ 33“ N, 0° 51′ 14′′ O) | adult | K, F: E, H | + | nd | + | + | PCR |
| 2014 | F 240 | France | Garonne | Golfech (44° 06′ 33“ N, 0° 51′ 14′′ O) | adult | K: E, H | + | Yes | + | + | PCR/CL |
| 2014 | F 241 | France | Garonne | Golfech (44° 06′ 33“ N, 0° 51′ 14′′ O) | adult | K, F: E, H | + | nd | + | + | PCR |
| 2014 | F 242 | France | Garonne | Golfech (44° 06′ 33“ N, 0° 51′ 14′′ O) | adult | K, F: E, H | + | nd | + | + | nd |
| 2014 | F 243 | France | Garonne | Golfech (44° 06′ 33“ N, 0° 51′ 14′′ O) | adult | K, F: E, H | + | nd | + | + | PCR/CL |
| 2014 | F 244 | France | Garonne | Golfech (44° 06′ 33“ N, 0° 51′ 14′′ O) | adult | K: E, H | + | YES | + | + | PCR |
| 2014 | F 245 | France | Garonne | Golfech (44° 06′ 33“ N, 0° 51′ 14′′ O) | adult | K: E, H | + | YES | + | + | nd |
| 2014 | F 246 | France | Garonne | Golfech (44° 06′ 33“ N, 0° 51′ 14′′ O) | adult | K: E, H | + | YES | + | + | nd |
| 2014 | F 247 | France | Garonne | Golfech (44° 06′ 33“ N, 0° 51′ 14′′ O) | adult | K: E, H | + | YES | + | + | nd |
| 2014 | F 248 | France | Garonne | Golfech (44° 06′ 33“ N, 0° 51′ 14′′ O) | adult | K: E, H | + | YES | + | + | nd |
| 2014 | F 249 | France | Garonne | Golfech (44° 06′ 33“ N, 0° 51′ 14′′ O) | adult | K: E, H | + | YES | + | + | nd |
| 2014 | F 250 | France | Garonne | Golfech (44° 06′ 33“ N, 0° 51′ 14′′ O) | adult | K: E, H | + | YES | + | + | nd |
| 2014 | F 251 | France | Garonne | Golfech (44° 06′ 33“ N, 0° 51′ 14′′ O) | adult | K: E, H | + | YES | + | + | nd |
| 2014 | F 252 | France | Garonne | Golfech (44° 06′ 33“ N, 0° 51′ 14′′ O) | adult | K: E, H | + | YES | nd | + | nd |
| 2014 | F 253 | France | Garonne | Golfech (44° 06′ 33“ N, 0° 51′ 14′′ O) | adult | K: E, H | + | YES | + | + | nd |
| 2014 | F 254 | France | Garonne | Golfech (44° 06′ 33“ N, 0° 51′ 14′′ O) | adult | K, F: E, H | + | nd | + | + | nd |
| 2014 | F 255 | France | Garonne | Golfech (44° 06′ 33“ N, 0° 51′ 14′′ O) | adult | K, F: E, H | + | nd | - | + | nd |
| 2014 | F 256 | France | Garonne | Golfech (44° 06′ 33“ N, 0° 51′ 14′′ O) | adult | K: E, H | + | YES | + | + | nd |
| 2014 | F 257 | France | Garonne | Golfech (44° 06′ 33“ N, 0° 51′ 14′′ O) | adult | K: E, H | + | YES | + | + | nd |
| 2014 | F 258 | France | Garonne | Golfech (44° 06′ 33“ N, 0° 51′ 14′′ O) | adult | K, F: E, H | - | nd | - | + | nd |
| 2014 | F 259 | France | Garonne | Golfech (44° 06′ 33“ N, 0° 51′ 14′′ O) | adult | K, F: E, H | - | nd | - | + | nd |
| 2014 | F 260 | France | Garonne | Golfech (44° 06′ 33“ N, 0° 51′ 14′′ O) | adult | K, F: E, H | - | nd | + | + | nd |
| 2014 | F 261 | France | Garonne | Golfech (44° 06′ 33“ N, 0° 51′ 14′′ O) | adult | K, F: E, H | - | nd | - | + | nd |
| 2014 | D 262 | Germany | Rhine | Urmitz (50°25'9.93"N; 7°31'9.49"E) | adult | K. F: E, H | - | nd | - | - | nd |
| 2014 | D 266 | Germany | Rhine | Ladenburg (49°28'34.80"N; 8°35'9.52"E) | adult | K: E, H | - | YES | + | - | nd |
| 2014 | D 268 | Germany | Rhine | Philippsburg (49° 4’ 9.3” N; 8° 25’ 55.2” E) | YOY | W, F: B | nd | nd | - | nd | nd |
| 2014 | D 269 | Germany | Rhine | Philippsburg (49° 4’ 9.3” N; 8° 25’ 55.2” E) | YOY | W, F: B | nd | nd | - | nd | nd |
| 2014 | D 270 | Germany | Rhine | Philippsburg (49° 4’ 9.3” N; 8° 25’ 55.2” E) | YOY | W, F: B | nd | nd | - | nd | nd |
| 2014 | D 271 | Germany | Rhine | Philippsburg (49° 4’ 9.3” N; 8° 25’ 55.2” E) | YOY | W, F: B | nd | nd | - | nd | nd |
| 2014 | D 272 | Germany | Rhine | Philippsburg (49° 4’ 9.3” N; 8° 25’ 55.2” E) | YOY | W, F: B | nd | nd | - | nd | nd |
| 2014 | D 273 | Germany | Rhine | Philippsburg (49° 4’ 9.3” N; 8° 25’ 55.2” E) | YOY | W, F: B | nd | nd | - | nd | nd |
| 2014 | D 274 | Germany | Rhine | Philippsburg (49° 4’ 9.3” N; 8° 25’ 55.2” E) | YOY | W, F: B | nd | nd | - | nd | nd |
| 2014 | D 275 | Germany | Rhine | Philippsburg (49° 4’ 9.3” N; 8° 25’ 55.2” E) | YOY | W, F: B | nd | nd | - | nd | nd |
| 2014 | D 276 | Germany | Rhine | Philippsburg (49° 4’ 9.3” N; 8° 25’ 55.2” E) | YOY | W, F: B | nd | nd | - | nd | nd |
| 2014 | D 277 | Germany | Rhine | Philippsburg (49° 4’ 9.3” N; 8° 25’ 55.2” E) | YOY | W, F: B | nd | nd | - | nd | nd |
| 2014 | D 278 | Germany | Rhine | Philippsburg (49° 4’ 9.3” N; 8° 25’ 55.2” E) | YOY | K, F: E | nd | nd | nd | - | nd |
| 2014 | D 279 | Germany | Rhine | Philippsburg (49° 4’ 9.3” N; 8° 25’ 55.2” E) | YOY | K, F: E | nd | nd | nd | - | nd |
| 2014 | D 280 | Germany | Rhine | Philippsburg (49° 4’ 9.3” N; 8° 25’ 55.2” E) | YOY | K, F: E | nd | nd | nd | - | nd |
| 2014 | D 281 | Germany | Rhine | Philippsburg (49° 4’ 9.3” N; 8° 25’ 55.2” E) | adult | K, F: E, H | - | nd | - | + | PCR/CL |
| 2014 | D 282 | Germany | Rhine | Philippsburg (49° 4’ 9.3” N; 8° 25’ 55.2” E) | YOY | K. F: E | nd | nd | nd | - | nd |
| 2014 | D 283 | Germany | Rhine | Philippsburg (49° 4’ 9.3” N; 8° 25’ 55.2” E) | YOY | K. F: E | nd | nd | nd | - | nd |
| 2014 | D 284 | Germany | Rhine | Kalkar Grieth (51°47'00.9"N 6°19'15.5"E) | YOY | K. F: E | nd | nd | nd | - | nd |
| 2014 | D 285 | Germany | Rhine | Kalkar Grieth (51°47'00.9"N 6°19'15.5"E) | YOY | K. F: E | nd | nd | nd | - | nd |
| 2014 | D 286 | Germany | Rhine | Kalkar Grieth (51°47'00.9"N 6°19'15.5"E) | YOY | K. F: E | nd | nd | nd | - | nd |
| 2014 | D 287 | Germany | Rhine | Kalkar Grieth (51°47'00.9"N 6°19'15.5"E) | YOY | K. F: E | nd | nd | nd | - | nd |
| 2014 | D 288 | Germany | Rhine | Kalkar Grieth (51°47'00.9"N 6°19'15.5"E) | YOY | K. F: E | nd | nd | nd | - | nd |
| 2014 | D 289 | Germany | Rhine | Kalkar Grieth (51°47'00.9"N 6°19'15.5"E) | YOY | K. F: E | nd | nd | nd | - | nd |
| 2014 | D 290 | Germany | Rhine | Speyer (49°24'19.20"N; 8°29'40.25"E) | adult | K, F: E, H | - | nd | - | + | PCR/CL |
| 2014 | D 291 | Germany | Rhine | Philippsburg (49° 4’ 9.3” N; 8° 25’ 55.2” E) | YOY | W, F: B | nd | nd | - | nd | nd |
| 2014 | D 292 | Germany | Rhine | Philippsburg (49° 4’ 9.3” N; 8° 25’ 55.2” E) | YOY | W, F: B | nd | nd | - | nd | nd |
| 2014 | D 293 | Germany | Rhine | Philippsburg (49° 4’ 9.3” N; 8° 25’ 55.2” E) | YOY | W, F: B | nd | nd | - | nd | nd |
| 2014 | D 294 | Germany | Rhine | Philippsburg (49° 4’ 9.3” N; 8° 25’ 55.2” E) | YOY | W, F: B | nd | nd | - | nd | nd |
| 2014 | D 295 | Germany | Rhine | Philippsburg (49° 4’ 9.3” N; 8° 25’ 55.2” E) | YOY | W, F: B | nd | nd | - | nd | nd |
| 2014 | D 296 | Germany | Rhine | Philippsburg (49° 4’ 9.3” N; 8° 25’ 55.2” E) | YOY | W, F: B | nd | nd | - | nd | nd |
| 2014 | D 297 | Germany | Rhine | Philippsburg (49° 4’ 9.3” N; 8° 25’ 55.2” E) | YOY | W, F: B | nd | nd | - | nd | nd |
| 2014 | D 298 | Germany | Rhine | Philippsburg (49° 4’ 9.3” N; 8° 25’ 55.2” E) | YOY | W, F: B | nd | nd | - | nd | nd |
| 2014 | D 299 | Germany | Rhine | Philippsburg (49° 4’ 9.3” N; 8° 25’ 55.2” E) | YOY | W, F: B | nd | nd | - | nd | nd |
| 2014 | D 300 | Germany | Rhine | Philippsburg (49° 4’ 9.3” N; 8° 25’ 55.2” E) | YOY | W, F: B | nd | nd | - | nd | nd |
| 2014 | D 301 | Germany | Rhine | Philippsburg (49° 4’ 9.3” N; 8° 25’ 55.2” E) | YOY | W, F: B | nd | nd | - | nd | nd |
| 2014 | D 302 | Germany | Rhine | Philippsburg (49° 4’ 9.3” N; 8° 25’ 55.2” E) | YOY | W, F: B | nd | nd | - | nd | nd |
| 2014 | D 303 | Germany | Rhine | Philippsburg (49° 4’ 9.3” N; 8° 25’ 55.2” E) | YOY | W, F: B | nd | nd | - | nd | nd |
| 2014 | D 304 | Germany | Rhine | Philippsburg (49° 4’ 9.3” N; 8° 25’ 55.2” E) | YOY | W, F: B | nd | nd | - | nd | nd |
| 2014 | D 305 | Germany | Rhine | Philippsburg (49° 4’ 9.3” N; 8° 25’ 55.2” E) | YOY | W, F: B | nd | nd | - | nd | nd |
| 2014 | D 306 | Germany | Rhine | Philippsburg (49° 4’ 9.3” N; 8° 25’ 55.2” E) | YOY | W, F: B | nd | nd | - | nd | nd |
| 2014 | D 307 | Germany | Rhine | Philippsburg (49° 4’ 9.3” N; 8° 25’ 55.2” E) | YOY | W, F: B | nd | nd | - | nd | nd |
| 2014 | D 308 | Germany | Rhine | Philippsburg (49° 4’ 9.3” N; 8° 25’ 55.2” E) | YOY | K: E | nd | nd | nd | - | nd |
| 2014 | D 309 | Germany | Rhine | Philippsburg (49° 4’ 9.3” N; 8° 25’ 55.2” E) | YOY | K: E | nd | nd | nd | - | nd |
| 2014 | D 310 | Germany | Rhine | Philippsburg (49° 4’ 9.3” N; 8° 25’ 55.2” E) | YOY | K: E | nd | nd | nd | - | nd |
| 2014 | D 311 | Germany | Rhine | Philippsburg (49° 4’ 9.3” N; 8° 25’ 55.2” E) | YOY | K: E | nd | nd | nd | - | nd |
| 2014 | D 312 | Germany | Rhine | Philippsburg (49° 4’ 9.3” N; 8° 25’ 55.2” E) | YOY | K: E | nd | nd | nd | - | nd |
| 2014 | D 314 | Germany | Rhine | Philippsburg (49° 4’ 9.3” N; 8° 25’ 55.2” E) | YOY | K: E | nd | nd | nd | - | nd |
| 2014 | D 315 | Germany | Rhine | Philippsburg (49° 4’ 9.3” N; 8° 25’ 55.2” E) | YOY | K: E | nd | nd | nd | - | nd |
| 2014 | D 316 | Germany | Rhine | Philippsburg (49° 4’ 9.3” N; 8° 25’ 55.2” E) | YOY | K: E | nd | nd | nd | - | nd |
| 2014 | D 317 | Germany | Rhine | Philippsburg (49° 4’ 9.3” N; 8° 25’ 55.2” E) | YOY | K: E | nd | nd | nd | - | nd |
| 2014 | D 318 | Germany | Rhine | Philippsburg (49° 4’ 9.3” N; 8° 25’ 55.2” E) | YOY | K: E | nd | nd | nd | - | nd |
| 2014 | D 319 | Germany | Rhine | Philippsburg (49° 4’ 9.3” N; 8° 25’ 55.2” E) | YOY | K: E | nd | nd | nd | - | nd |
| 2014 | D 320 | Germany | Rhine | Philippsburg (49° 4’ 9.3” N; 8° 25’ 55.2” E) | YOY | K: E | nd | nd | nd | - | nd |
| 2014 | D 321 | Germany | Rhine | Philippsburg (49° 4’ 9.3” N; 8° 25’ 55.2” E) | YOY | K: E | nd | nd | nd | - | nd |
| 2014 | D 322 | Germany | Rhine | Philippsburg (49° 4’ 9.3” N; 8° 25’ 55.2” E) | YOY | K: E | nd | nd | nd | - | nd |
| 2014 | D 323 | Germany | Rhine | Philippsburg (49° 4’ 9.3” N; 8° 25’ 55.2” E) | YOY | K: E | nd | nd | nd | - | nd |
| 2014 | F 334 | France | Garonne | Couthures | YOY | K: E | nd | nd | nd | + | nd |
| 2014 | F 335 | France | Garonne | Sainte-Bazeille | YOY | K: E | nd | nd | nd | - | nd |
| 2014 | F 336 | France | Garonne | Couthures | YOY | K: E | nd | nd | nd | - | nd |
| 2014 | F 337 | France | Garonne | Couthures | YOY | K: E | nd | nd | nd | - | nd |
| 2014 | F 338 | France | Garonne | Marmande | YOY | K: E | nd | nd | nd | + | nd |
| 2014 | F 339 | France | Garonne | Marmande | YOY | K: E | nd | nd | nd | + | nd |
| 2014 | F 340 | France | Garonne | Sainte-Bazeille | YOY | K: E | nd | nd | nd | + | nd |
| 2014 | F 341 | France | Garonne | Couthures | YOY | K: E | nd | nd | nd | + | PCR/CL |
| 2014 | F 342 | France | Garonne | Couthures | YOY | K: E | nd | nd | nd | + | nd |
| 2014 | F 343 | France | Garonne | Couthures | YOY | K: E | nd | nd | nd | - | nd |
| 2014 | F 344 | France | Garonne | Marmande | YOY | K: E | nd | nd | nd | - | nd |
| 2014 | F 345 | France | Garonne | Marmande | YOY | K: E | nd | nd | nd | + | nd |
| 2014 | F 346 | France | Garonne | Sainte-Bazeille | YOY | K: E | nd | nd | nd | + | nd |
| 2014 | F 347 | France | Garonne | Couthures | YOY | K: E | nd | nd | nd | + | PCR/CL |
| 2014 | F 348 | France | Garonne | Couthures | YOY | K: E | nd | nd | nd | + | PCR/CL |
| 2014 | F 349 | France | Garonne | Sainte-Bazeille | YOY | K: E | nd | nd | nd | + | nd |
| 2014 | F 350 | France | Garonne | Sainte-Bazeille | YOY | K: E | nd | nd | nd | - | nd |
| 2014 | F 351 | France | Garonne | Sainte-Bazeille | YOY | K: E | nd | nd | nd | - | nd |
| 2014 | F 352 | France | Garonne | Couthures | YOY | K: E | nd | nd | nd | - | nd |
