# Supplementary material for: Repatriation of an old fish host as an opportunity for myxozoan parasite diversity: The example of the allis shad, Alosa alosa (Clupeidae), in the Rhine
Source: Parasit Vectors. 2016 Sep 15;9:505. doi: 10.1186/s13071-016-1760-6 (PMC5024467; doi:10.1186/s13071-016-1760-6)
Supplement: Additional file 2: Table S2. — SSU rDNA variability of Hoferellus alosae n. sp. clones from fish individuals in the Garonne. (DOCX 18 kb) [file 13071_2016_1760_MOESM2_ESM.docx]

**Additional file 3: Table S2**. SSU rDNA variability of *Hoferellus alosae* n. sp. clones from fish individuals in the **Garonne**. Six clones of 901 bp were sequenced from each fish individual.

| **Adults**  Position in the alignment | Base change | Change frequency |
| --- | --- | --- |
| **Fish individual 39 –> 5/901** |  |  |
| 504  **655**  737  763  830 | G(A)  **C(T)**  G(A)  T(C)  G(A) | 1/6  **1/6**  1/6  1/6  1/6 |

**Fish individual F240 –> 3/901**

| 584  **655**  895 | C(T)  **C(T)**  G(A) | 1/6  **1/6**  1/6 |
| --- | --- | --- |

**Fish individual F243 –> 5/901**

| 7  60  91  184  **655** | A(G)  A(G)  C(T)  C(T)  **C(T)** | 1/6  1/6  1/6  1/6  **1/6** |
| --- | --- | --- |
| **Young-of-the-year**  Position in the alignment | Base change | Change frequency |
| **Fish individual F241 –> 7/901** |  |  |
| 15  137  290  617  **655**  695  876 | G(A)  C(T)  T(C)  G(A)  **C(T)**  G(A)  G(A) | 1/6  1/6  1/6  1/6  **1/6**  1/6  1/6 |

**Fish individual F247 –> 3/901**

| 895  441  756 | G(A)  G(A)  C(T) | 1/6  1/6  1/6 |
| --- | --- | --- |

**Fish individual F248 –> 5/901**

| 34  183  402  **655**  797 | T(G)  A(G)  T(C)  **C(T)**  T(C) | 1/6  1/6  1/6  **2/6**  1/6 |
| --- | --- | --- |

Note: Base changes in red colour mark polymorphic sites.
