# Supplementary material for: Repatriation of an old fish host as an opportunity for myxozoan parasite diversity: The example of the allis shad, Alosa alosa (Clupeidae), in the Rhine
Source: Parasit Vectors. 2016 Sep 15;9:505. doi: 10.1186/s13071-016-1760-6 (PMC5024467; doi:10.1186/s13071-016-1760-6)
Supplement: Additional file 3: Table S3. — SSU rDNA variability of Hoferellus alosae n. sp. clones from fish individuals in the Dordogne. (DOCX 17 kb) [file 13071_2016_1760_MOESM3_ESM.docx]

**Additional file 4: Table S3.** SSU rDNA variability of *Hoferellus alosae* n. sp. clones from fish individuals in the **Dordogne**. Six clones of 901 bp were sequenced from each fish individual.

| Position in the alignment | Base change | Change frequency |
| --- | --- | --- |
| **Fish individual 179 –> 10/901** |  |  |
| **95**  **519**  **522**  542  **585**  **603**  762  783  816  **820** | **A(G)**  **T(C)**  **T(C)**  A(G)  **A(G)**  **C(T)**  G(A)  C(T)  G(A)  **C(T)** | **6/6**  **6/6**  **6/6**  1/6  **6/6**  **6/6**  1/6  1/6  1/6  **6/6** |

**Fish individual 187 –>9/901**

| **95**  129  477  **519**  **522**  546  **585**  **603**  **820** | **A(G)**  C(T)  G(A)  **T(C)**  **T(C)**  C(T)  **A(G)**  **C(T)**  **C(T)** | **1/6**  1/6  1/6  **1/6**  **1/6**  1/6  **1/6**  **1/6**  **2/6** |
| --- | --- | --- |

**Fish individual 188 –> 9/901**

| **95**  394  368  482  **519**  **522**  **585**  **603**  **820** | **A(G)**  C(T)  G(A)  C(T)  **T(C)**  **T(C)**  **A(G)**  **C(T)**  **C(T)** | **3/6**  1/6  1/6  1/6  **5/6**  **5/6**  **2/6**  **5/6**  **5/6** |
| --- | --- | --- |

Note: Base changes in red colour mark polymorphic sites.
