# Supplementary material for: Repatriation of an old fish host as an opportunity for myxozoan parasite diversity: The example of the allis shad, Alosa alosa (Clupeidae), in the Rhine
Source: Parasit Vectors. 2016 Sep 15;9:505. doi: 10.1186/s13071-016-1760-6 (PMC5024467; doi:10.1186/s13071-016-1760-6)
Supplement: Additional file 4: Table S4. — SSU rDNA variability of Hoferellus alosae n. sp. clones from fish individuals in the Rhine. (DOCX 16 kb) [file 13071_2016_1760_MOESM4_ESM.docx]

**Additional file 5: Table S4.** SSU rDNA variability of *Hoferellus alosae* n. sp. clones from fish individuals in the **Rhine**. Six clones of 901 bp were sequenced from each fish individual.

| Position in the alignment | Base change | Change frequency |
| --- | --- | --- |

**Fish individual D281 –> 15/901**

| **91**  108  225  260  262  298  309  575  648  674  682  765  819  829  899 | **C(T)**  A(T)  C(T)  G(A)  T(C)  G(A)  A(G)  C(T)  C(T)  G(T)  G(A)  C(T)  A(G)  G(A)  C(T) | **3/6**  1/6  1/6  1/6  1/6  1/6  1/6  1/6  1/6  1/6  1/6  1/6  1/6  1/6  1/6 |
| --- | --- | --- |

**Fish individual D290 –> 18/901**

| 26  **91**  207  219  310  368  433  452  461  468  544  **655**  678  684  697  763  799  851 | C(T)  **C(T)**  G(A)  A(G)  G(A)  G(A)  T(G)  C(T)  C(A)  C(T)  A(G)  **C(T)**  G(A)  G(T)  G(A)  T(C)  A(G)  C(T) | 1/6  **1/6**  1/6  1/6  1/6  1/6  1/6  1/6  1/6  1/6  1/6  **6/6**  1/6  1/6  1/6  1/6  1/6  1/6 |
| --- | --- | --- |

Note: Base changes in red colour mark polymorphic sites.
