# Supplementary figures and images for: Repatriation of an old fish host as an opportunity for myxozoan parasite diversity: The example of the allis shad, Alosa alosa (Clupeidae), in the Rhine
Source: Parasit Vectors. 2016 Sep 15;9:505. doi: 10.1186/s13071-016-1760-6 (PMC5024467; doi:10.1186/s13071-016-1760-6)

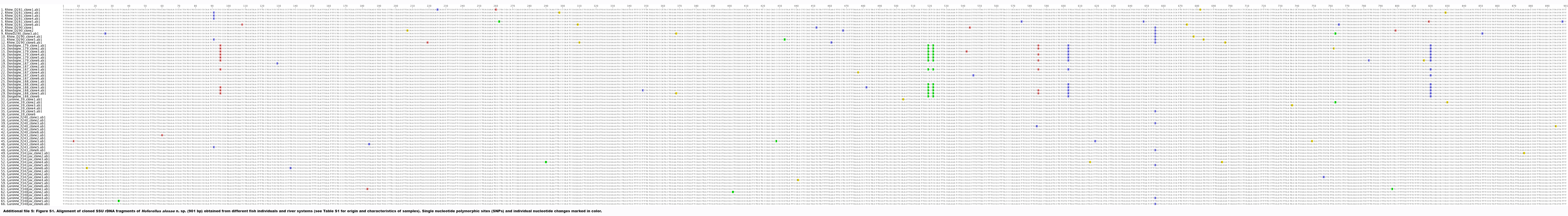

Supplement: Additional file 5: Figure S1. — Alignment of cloned SSU rDNA fragments of Hoferellus alosae n. sp. (901 bp) obtained from different fish individuals and river systems (see Additional file 1: Table S1 for origin and characteristics of samples). Single nucleotide polymorphic sites (SNPs) and individual nucleotide changes marked in color. (PNG 557 kb) [file 13071_2016_1760_MOESM5_ESM.png]
